# Assessment of Knowledge, Stigmatizing Attitudes and Health-Seeking Behaviors Regarding Hepatitis B Virus Infection in a Pharmacy and Community Setting in Sierra Leone: A Cross-Sectional Study

**DOI:** 10.3390/healthcare11020177

**Published:** 2023-01-06

**Authors:** Manal Ghazzawi, Sahr A. Yendewa, Peter B. James, Samuel P. Massaquoi, Lawrence S. Babawo, Foday Sahr, Gibrilla F. Deen, Mustapha Kabba, Ponsiano Ocama, Sulaiman Lakoh, Robert A. Salata, George A. Yendewa

**Affiliations:** 1 KnowHep Foundation, Freetown, Sierra Leone; 2 Ministry of Health and Sanitation, Freetown, Sierra Leone; 3 Faculty of Health, Southern Cross University, Lismore, NSW 2480, Australia; 4 Department of Nursing, School of Community Health Sciences, Njala University, Bo Campus, Freetown, Sierra Leone; 5 Department of Medicine, College of Medicine and Allied Health Sciences, University of Sierra Leone, Freetown, Sierra Leone; 6 Department of Medicine, College of Health Sciences, Makerere University, Kampala P.O. Box 7062, Uganda; 7 Department of Medicine, Case Western Reserve University School of Medicine, Cleveland, OH 44106, USA; 8 Division of Infectious Diseases and HIV Medicine, University Hospitals Cleveland Medical Center, Cleveland, OH 44106, USA; 9 Johns Hopkins Bloomberg School of Public Health, Baltimore, MD 21205, USA

**Keywords:** hepatitis B virus, knowledge, stigma, health-seeking behaviors, Sierra Leone

## Abstract

Hepatitis B virus (HBV) is a major global health challenge. Emerging evidence suggests that poor knowledge and stigma are impacting HBV control efforts in sub-Saharan Africa (SSA), but their role is not well understood. We conducted a cross-sectional study of adults aged ≥18 years in a community and pharmacy setting in Freetown, Sierra Leone. A structured questionnaire was used to assess knowledge, stigmatizing attitudes and health-seeking behaviors regarding HBV. Logistic regression was used to identify predictors of HBV knowledge and related stigma. A total of 306 adult participants were enrolled (50.7% male, 7.5% HBV positive and 11.7% vaccinated). Overall, 52.2% had good HBV knowledge and 49.3% expressed a stigmatizing attitude towards people with HBV. Notwithstanding, 72.2% stated they would receive the HBV vaccine if offered, 80.4% would take anti-HBV medication and 78.8% would be willing to attend clinic regularly. Good HBV knowledge was associated with HBV positive status (aOR 4.41; *p* = 0.029) and being vaccinated against HBV (aOR 3.30; *p* = 0.034). HBV-related stigma was associated with secondary or higher level of education (aOR 2.36; *p* < 0.001), good HBV knowledge (aOR 2.05; *p* = 0.006) and pharmacy setting (aOR 1.74, *p* = 0.037). These findings suggest that education and stigma reduction may benefit HBV elimination efforts in SSA.

## 1. Introduction

Hepatitis B virus (HBV) infection is a major global health concern. According to World Organization (WHO) estimates, there were 296 million chronic cases of HBV globally in 2019, most of which remain undiagnosed or untreated [1]. People with chronic HBV are at increased risk of developing serious clinical sequelae including liver cirrhosis and hepatocellular carcinoma, which together accounted for 820,000 deaths in 2019, surpassing deaths caused by the human immunodeficiency virus (HIV) epidemic in the same year [1]. Communities in sub-Saharan Africa (SSA) are disproportionately impacted by the HBV epidemic, with countries in the WHO West and Central Africa region reporting some of the highest prevalence rates of HBV globally (prevalence > 8%) [1,2,3]. Sierra Leone is regarded as a hyperendemic country, with several recent studies recording HBV prevalence rates ranging from 8% to 22% in various demographic groups across the country [4,5,6,7].

Owing to the magnitude of its global impact, the WHO Global Health Sector Strategy has proposed a roadmap for the elimination of viral hepatitis as a public health threat by 2030, defined as a 90% reduction in new cases and 65% reduction in mortality compared with the 2015 baseline [8]. Sierra Leone and other countries in SSA with high HBV endemicity are in the process of implementing national strategies for scaling up prevention and control of HBV but are constrained by limited resources in the setting of myriad other competing developmental challenges [2,8,9]. Additionally, there is emerging evidence that barriers such as limited knowledge of HBV in the general population [9,10], stigmatizing attitudes towards people living with HBV [11,12] and high levels of poverty [10] could be influencing willingness to engage with HBV screening, treatment and prevention efforts in SSA. Hence, dispelling negative perceptions around HBV through educational outreach as well as addressing social conditions that perpetuate the cycles of poverty and underdevelopment should be an integral part of ongoing HBV elimination efforts in such resource-limited settings [9,10,11,12].

Despite the high prevalence of HBV in Sierra Leone, there is limited evidence describing perceptions, attitudes and practices regarding HBV to help inform policy or optimize public health interventions. In a 2017 study by Qin et al. [13], 34.6% and 6.6% of healthcare workers surveyed at a tertiary level hospital in Sierra Leone had good knowledge of the routes of transmission and clinical outcomes of HBV infection, respectively. In another study of healthcare workers from Sierra Leone, Massaquoi et al. [14] reported that only 11.7% of study participants had adequate knowledge of the epidemiology and modes of transmission of HBV. However, no studies have comprehensively explored these factors in the general population. In this study, we aimed to examine the level of knowledge, health-seeking behaviors and stigmatizing attitudes regarding HBV in a pharmacy and community settings in Freetown, Sierra Leone. Additionally, we aimed to assess the associated factors of good HBV knowledge and stigma expressed towards people living with HBV in this setting.

## 2. Materials and Methods

### 2.1. Study Sites and Context

The study was conducted in collaboration with KnowHep Foundation, a non-profit organization based in Freetown, the capital city of Sierra Leone. The population of Freetown is estimated at 1.2 million and the city is divided into eastern, central and western districts. KnowHep Foundation was established in 2019, with the goal of increasing awareness of viral hepatitis through advocacy and educational outreach activities in Freetown and the rest of the country. There are few specialty clinics dedicated to the care of patients with HBV in Sierra Leone. To address this gap in service delivery, KnowHep Foundation provides free or low-cost HBV services in Freetown through CitiGlobe Pharmacies Ltd., an affiliated network of three community-based pharmacies. Services offered include HBV screening, HBV vaccination and referrals for clinic-based HBV care. KnowHep Foundation’s community outreach activities are facilitated through partnerships with various civil society organizations in Freetown including women’s groups, youth empowerment groups and student organizations. Since 2019, community outreach activities involving HBV screening, vaccination and raising awareness of HBV are conducted at least four times yearly in Freetown, including on World Hepatitis Day (every 28 July).

### 2.2. Study Design, Period, Population and Recruitment

We conducted a cross-sectional study of adults aged ≥18 years from January to July 2022 to assess their level of knowledge of HBV, stigmatizing attitudes towards people living with HBV and health-seeking behaviors regarding HBV. Convenience sampling was employed to sequentially enroll participants in both the pharmacy and community settings by trained research staff. The CitiGlobe Pharmacies Ltd. branch in the Western district of Freetown was used for the pharmacy-based part of the study, as it is the largest branch in the pharmacy network, with about 50 to 70 pharmacy customer visits daily. On the other hand, the community outreach-based part of the study was conducted during outreach activities, as described above. Regular customers coming to the pharmacy or members of the general public participating in our community outreach activities were approached by research staff and informed about the study. In each setting, those who expressed interest in the study were enrolled after providing written informed consent.

### 2.3. Sample Size Determination and Justification

We used the formula according to Lwanga and Lemeshow [15] to estimate the sample size, n as follows:

n = Z^2^ × *p* (1 **−**
*p*)/e^2^

Massaquoi et al. [14] have conducted the most comprehensive study to date in Sierra Leone, with 11.7% of healthcare workers surveyed demonstrating adequate knowledge of the epidemiology and modes of transmission of HBV. Assuming *p* = 0.117, 95% confidence interval (Z = 1.96) and a 5% margin of error (e = 0.05) would yield a minimum sample size of 159, assuming no association under the null hypothesis using a 2-tailed test with a significance level of 0.05.

### 2.4. Survey Instrument Development and Validation Process

The survey instrument was composed of two sections. The first section entailed a questionnaire on sociodemographic and health-related information including history of HBV infection, vaccination status and recognized risk factors for HBV infection.

The second section initially entailed a 38-item structured questionnaire adapted from relevant studies in the literature [13,14,16,17,18,19,20,21,22,23,24] which tested survey respondents in the three HBV competencies as follows: i.e., knowledge of HBV, attitudes towards HBV and preventive practices and health-seeking behaviors (Supplementary Materials). Each item had three response options: “Yes”, “No” and “I don’t know”. The questionnaire was pre-tested on a sample of respondents (n = 20) to assess understandability and clarity. Items were removed which were deemed ambiguous, repetitive, too difficult or garnered a response rate of less than 50%. The final survey version of the questionnaire consisted of 33 items structured as follows: (1) Part 1, on HBV Knowledge, which was further categorized into HBV epidemiology (4 items), modes of transmission (9 items), symptoms and sequelae (5 items) and prevention and treatment (10 items); and Part 2, which was further categorized into health-seeking behaviors (3 items) and stigmatizing attitudes towards people living with HBV (2 items).

### 2.5. Data Collection, Scoring and Definitions of Study Measures

The survey was administered to participants by trained research staff. Participants who could read and write filled out the questionnaires by themselves, with research staff helping clarify any misunderstands that arose. Those who could not read or write had the survey read out to them by the research staff. All returned questionnaires were examined for completeness, consistency and clarity before entry into a spreadsheet. Sociodemographic information collected included age, gender, relationship status, highest education attained, employment status and monthly earnings. Tertiary education was defined as any post-secondary education. Formal sector employment included any job with regular wages and benefits, while informal sector jobs included petty trading, subsistence farming and seasonal jobs. In line with current salary structure and cost of living in Sierra Leone, low earning was defined as <100 USD/month and high earning was defined as ≥100 USD/month.

Each item on the questionnaire answered correctly earned 1 point. To estimate HBV knowledge levels, knowledge scores were constructed by summing all the responses of participants on the knowledge part of the survey (Part 1), with a mininum possible score of 0 and maximum possible score of 28. Given that this was a lay population which was not expected to have a high baseline HBV knowledge level in comparison with healthcare workers (e.g., doctors, nurses, medical students), we defined two categories of HBV knowledge level, as follows: (1) good or acceptable level of HBV knowledge, defined as scoring ≥ mean HBV knowledge score (i.e., approximately 50th percentile), while scoring < mean HBV knowledge score was regarded as poor HBV knowledge; and (2) comprehensive HBV knowledge, defined as scoring ≥ 75th knowledge score. Stigmatizing attitude towards people with HBV was defined as harboring at least one of the two negative attitudes tested in the survey and earned 1 point. Similarly, items regarding health-seeking behaviors which were answered in the affirmative were rated as positive attitudes and earned 1 point.

### 2.6. Statistical Analysis

Statistical analyses were performed using the SPSS Version 28.0 (IBM Corp, Armonk, NY, USA). Categorical variables were reported as frequencies (percentages) and compared using Pearson’s chi-square or Fisher’s exact tests. Continuous variables were presented as means (standard deviation) or median (range) and compared using the non-parametric independent samples Mann–Whitney U-test or Kruskal–Wallis test, as appropriate. Logistic regression was used to identify predictors of good HBV knowledge and expressed stigma towards people with HBV. Variables that attained a *p*-value of <0.2 in the univariate analysis were included in the final multivariate regression model. Associations were reported as crude (OR) and adjusted odds ratios (aOR) with 95% confidence intervals (CI). In all analyses, differences were considered statistically significant when *p* was <0.05.

### 2.7. Ethical Approval

Ethical approval was obtained from the Sierra Leone Ethics and Scientific Review Committee (approval date 21 December 2021). Prior to enrolment, written informed consent was obtained from the participants. Participation was strictly voluntary and the participants could withdraw from the study at any stage.

## 3. Results

### 3.1. Characteristics of Study Participants

Overall, 306 participants were enrolled in the study, 54.6% through community (non-healthcare) outreach activities and 45.4% through pharmacy (healthcare)-based outreach. Table 1 describes the sociodemographic and health characteristics of the study participants. About half were male (50.7%), 41.5% were aged 25–34 years, 66.0% were single and most (87.6%) had attained at least primary level of education. Over half were unemployed (53.9%) and 47.4% resided in the Western part of Freetown. The majority (58.2%) had never tested for HBV, while 7.5% disclosed that they had had HBV. The self-reported HBV vaccination rate was low, at 11.1%, and 8.8% reported having a family member, friend or colleague with HBV.

### 3.2. Assessment of HBV Knowledge

A total of 28 questions assessed HBV knowledge under the categories of epidemiology, modes of transmission, symptoms and sequelae and prevention and treatment. Table 2 describes the proportion of correct responses to survey questions, while Table 3 presents the mean and median HBV knowledge scores and the proportion of participants who scored above the mean (i.e., had good HBV knowledge) in each category.

The overall mean HBV knowledge score was 14.1 ± 8.0, with 52.2% of study participants demonstrating good knowledge of HBV (Table 3), while 25.2% (77 participants) scored ≥ 21 (i.e., 75th percentile) and were categorized as having comprehensive knowledge of HBV. Within HBV knowledge categories, about 60.0% of participants had good knowledge of HBV epidemiology category (mean score 2.4 ± 1.4) (Table 3). The majority of study participants (74.5%) correctly stated that both men and women are affected by HBV; however, less than half (46.4%) knew that HBV is caused by a virus, while over two-thirds (67.6%) believed HBV infection is caused by a curse or evil spirits. Despite Sierra Leone being highly endemic for HBV, under half of study participants (45.4%) were aware that HBV was common in the country.

Over half (54.4%) had good understanding of the modes of HBV transmission category (mean score 4.9 ± 2.8) (Table 3). Most correctly identified transfusion of contaminated blood (65.7%), unprotected sex with HBV-infected partner (65.0%) and injury from contaminated needles or sharps (66.3%) as modes of HBV transmission. However, less than half knew that HBV can be transmitted from mother to child (48.0%). Notwithstanding, the majority knew that HBV is not spread by shaking hands (80.7%) and eating or sharing food and utensils (66.3%). Additionally, a minority knew HBV cannot transmitted though mosquito or insect bites (43.8%), or through coughing or sneezing (34.6%).

In the symptoms and sequelae category, about 46% had good knowledge, with mean score 2.3 ± 2.0 (Table 2). Less than half (43.8%) knew that most people with HBV present without symptoms, while 54.6% were aware of scleral jaundice as a feature of HBV infection. Nearly half (47.7%) correctly identified liver cancer as a complication of HBV infection.

The mean prevention and treatment category score was 4.6 ± 2.9, with 47% of participants overall demonstrating good knowledge of this category (Table 3). Over one-third (34.6%) were aware of a safe and effective vaccine against HBV, while 41.5% and 45.8% knew that vaccinating pregnant women and newborns, respectively, protects against HBV. The majority understood that HBV can be prevented through screening of blood before transfusion (65.0%) and appropriate condom use (64.1%) can protect against HBV acquisition. Similarly, most (64.7%) believed that HBV is not curable but can be effectively managed with medications. Interestingly, half of the participants (50.3%) also believed that HBV can be treated with herbs or traditional medicines.

### 3.3. Assessment of Stigmatizing Attitudes towards People with HBV

Of the two items assessing stigmatizing attitudes (Table 2), 43.5% of the participants had concerns with sharing food or utensils with someone with HBV. Similarly, 44.1% stated that they would have concerns with having casual contact or working with a person known to have HBV. Overall, nearly half (49.3%) expressed at least one stigmatizing attitude towards people with HBV.

### 3.4. Assessment of Health-Seeking Behaviors

Three questions were tested to assess health-seeking behaviors towards HBV (Table 2). The responses were overwhelmingly positive. The majority stated that they would receive the HBV vaccine of offered for free (72.2%). Furthermore, 80.4% were willing to take medication for treatment if they tested positive for HBV, while a similar proportion (78.8%) were willing to undergo regular clinic follow up every 3 to 6 months for the management of HBV.

### 3.5. Factors Associated with Good HBV Knowledge

Table 4 shows the associations between good HBV knowledge and sociodemographic variables. In the multivariate analysis, participants with HBV (aOR 4.41, 95% CI [1.16–16.77]; *p* = 0.029) and those who are vaccinated against HBV (aOR 3.30, 95% CI [1.09–9.94]; *p* = 0.034) were independently associated with good HBV knowledge.

### 3.6. Factors Associated with HBV-Related Stigma

As summarized in Table 5, HBV-related stigma was associated with having a secondary or higher level of education (aOR 2.36, 95% CI [1.20–4.66]; *p* < 0.001), having good HBV knowledge (aOR 2.05, 95% CI [1.23–3.43]; *p* = 0.006) and participants who were recruited in the pharmacy setting (aOR 1.74, 95%CI [1.03–2.93]; *p* = 0.037).

## 4. Discussion

In line with the 2030 global viral hepatitis elimination goals, there has been increasing recognition in Sierra Leone in recent years that the HBV epidemic needs tackling urgently. Accordingly, several studies have attempted to address the prevalence and associated factors of HBV infection in various demographic groups in Sierra Leone. However, there is a dearth of research examining knowledge, attitudes and practices regarding the disease in the country and what limited evidence exists has focused on healthcare workers as a high-risk group due to the increased likelihood of occupational exposure to HBV [13,14]. To the best of our knowledge, this is the first study from Sierra Leone to comprehensively evaluate knowledge, stigmatizing attitudes and health-seeking behaviors regarding HBV infection in the general population. As this study was largely formative, we sequentially enrolled a cross-section of members of the general public during our educational outreach and advocacy activities in a non-healthcare (community) and healthcare (pharmacy) setting in Freetown, the capital and largest city of Sierra Leone.

Despite the fact that most (87.6%) study participants had received formal education (i.e., attained primary education or higher), just over half (52.2%) demonstrated good knowledge of HBV. Studies from several countries across SSA have reported similarly low levels of HBV knowledge in both healthcare and non-healthcare settings [13,14,16,17,18,19]. These studies reveal a substantial knowledge gap in endemic countries and suggest that, unlike other common communicable diseases such as HIV, tuberculosis and malaria, the HBV epidemic may not be receiving sufficient attention in the educational curriculum and public dialogues taking place in civil society in Sierra Leone. This is reflected in the fact that less than half (46.4%) of study participants knew about the viral etiology of HBV, while two-thirds (67.6%) believed that HBV could be caused by supernatural forces. Additionally, less than 50% demonstrated adequate knowledge of the modes of transmission, clinical signs and symptoms and serious sequalae of HBV infection such as cirrhosis and liver cancer, while only one-third (34.6%) were aware of a safe and effective vaccine for the prevention of HBV infection. These findings indicate a need to intensify efforts towards awareness-raising and advocacy around HBV in institutions of learning as well as in the public domain in Sierra Leone.

Contrary to findings from previous studies from other endemic countries [13,17,22,23,24], level of knowledge of HBV was not associated with age, education, relationship status, income, or survey setting (healthcare versus non-healthcare). However, survey participants with HBV positive status and those who had received the HBV vaccine were significantly more likely to demonstrate good knowledge of HBV. These findings were not entirely unexpected and have been previously advanced by several theories of health which have posited that diagnosis with health conditions such as chronic HBV infection which require long-term engagement with the healthcare system can increase understanding of disease processes, which can in turn motivate positive health behavioral change, including willingness to get tested and immunized, better compliance with treatment plans and remaining engaged in care—all of which yield individual and societal health dividends [22,25]. An encouraging finding of our study was that, despite the low level of knowledge of HBV, the majority of survey participants (70% to 80%) were favorably disposed to HBV vaccination, taking anti-HBV medication and remaining engaged in HBV care with regular clinic follow-ups if required.

A key finding of our study was the emergence of HBV-related stigma as a potential barrier to health seeking and health outcomes in this setting. Alarmingly, nearly half (49.3%) of study participants expressed a stigmatizing attitude towards people living with HBV. Individual- and community-level stigma related to communicable diseases are widely recognized and have previously been described in Sierra Leone in the context of HIV and Ebola by Kelly et al. [26] and James et al. [27], respectively. In particular, stigma related to HIV can have long-lasting deleterious health consequences, including non-disclosure of status, delayed health seeking, disengagement from care, treatment non-adherence, increased risk of poor mental wellbeing and an overall depreciation in quality of life [28,29]. However, there is limited understanding of the nature and long-term health impacts of HBV-related stigma in endemic countries in SSA. A recent systematic review by Mokaya et al. [12] showed that only 2 of 32 studies on HBV-related stigma were from SSA [30,31]; most of the emerging evidence comes from studies conducted in Asia and immigrant-origin communities from HBV-endemic regions resettled in the United States and other high-income countries [32,33,34,35]. Collectively, these studies indicate that stigma is a burgeoning problem that is hampering the successful implementation of HBV programs, highlighting a need for systematic and rigorous inquiry into a nascent area of research [12].

Furthermore, our analysis identified secondary education or higher, having good knowledge of HBV and the healthcare (pharmacy) setting as independent predictors of HBV-related stigma. These findings reveal the complex nature of stigma as a social phenomenon and suggest that having accurate knowledge of disease processes and being sufficiently educated may not be enough to overcome stereotypes and negative perceptions of people affected by a communicable disease [25,28,29]. A plausible explanation is that more knowledge of a disease can paradoxically result in further entrenchment rather than dispelling of stigma; on the other hand, stigma may be rare in the setting of limited awareness of a disease. In a formative study from a low prevalence region in Uganda (HBV prevalence < 3%), Mugisha et al. [36] reported that there was no specific word for HBV infection in the local languages, which resulted in low levels of observed HBV-related stigma in a community that was largely unfamiliar with the concept. Similarly, where HBV-related stigma has been reported in the healthcare setting, it has been attributed mainly to poor knowledge among healthcare workers, which has served as an impediment to effective care delivery to people with HBV [36,37,38]. Given the widespread use of traditional, alternative and complementary medicine use in SSA [39], it is also likely that survey participants recruited in the pharmacy setting were more likely to be health literate in general and therefore more likely to be knowledgeable about conventional medicine and HBV disease process, paradoxically resulting in more expressed stigma towards people with HBV. The multidimensional nature of stigma in this setting therefore warrants larger population-based studies in an attempt to carefully define its prevalence and correlates as the first step towards synthesizing evidence-based interventions to aid HBV elimination efforts in Sierra Leone.

Our study had limitations and strengths. Firstly, we employed convenience sampling and a small sample size restricted to an urban setting in Freetown, which makes generalizability of results difficult. Secondly, we used close-ended questions, which did not allow respondents to provide more nuanced and informative insights into prevailing perceptions and attitudes towards HBV. Thirdly, the respondents were relatively young and educated, which may have skewed our findings. Lastly, HBV-related stigma was assessed using only two items, which makes it difficult to draw definitive conclusions; a more detailed exploration of the multiple dimensions of HBV-related stigma is therefore warranted to better understand this phenomenon. Despite these limitations, this formative study from Sierra Leone is the first to provide an in-depth analysis of a subject matter with critical public health implications and its findings will assist in planning more detailed studies which will be crucial in developing the evidence base needed to effectively tackle the HBV problem in Sierra Leone in line with the 2030 global viral hepatitis elimination goals.

## 5. Conclusions

In the first study from Sierra Leone to comprehensively assess knowledge, attitudes and health-seeking behaviors regarding HBV in the general population, we found that about half of all people surveyed had good knowledge of HBV, while simultaneously harboring stigmatizing attitudes towards people with the infection. Despite this, the majority were favorably disposed to receiving the HBV vaccine, treatment for HBV if indicated and remaining engaged in care by regularly attending clinic follow-up if required. Our findings suggest that interventions aimed at increasing HBV knowledge and reducing HBV-related stigma may benefit HBV elimination efforts in SSA.

## Figures and Tables

**Table 1:** Sociodemographic and clinical characteristics of study participants (N = 306).

Characteristics	N	%
**Gender**		
Male	155	50.7
Female	151	49.3
**Age, years**		
<25	94	30.7
25–34	127	41.5
35–44	44	14.4
≥45	41	13.4
**Relationship status**		
Single	202	66.0
Married	88	28.8
Divorced	4	1.3
Widowed	12	3.9
**Highest education attained**		
None	38	12.4
Primary	23	7.5
Secondary	73	23.9
Tertiary	172	56.2
**Employment status**		
Unemployed	165	53.9
Informal sector	87	28.4
Formal sector	54	17.6
**Monthly earnings**		
Low (<100 US dollars)	173	56.5
High (≥100 US dollars)	133	43.5
**HBV status**		
Positive	23	7.5
Negative	105	34.3
Never tested	178	58.2
**HBV vaccination status**		
Vaccinated	34	11.1
Not vaccinated	272	88.9
**Has a family member, friend or colleague with HBV**		
Yes	27	8.8
No	279	91.2
**Venue of HBV survey**		
Community outreach	167	54.6
Pharmacy-based	139	45.4

**Table 2:** Knowledge of HBV epidemiology, diagnosis and treatment.

Questions		Responses	
	**Epidemiology**	**Correct answer**	**%**
1	Hepatitis B is caused by a virus	142	46.4
2	Hepatitis B can be caused by a curse or evil spirits	207	67.6
3	Hepatitis B affects both males and females	228	74.5
4	Hepatitis B is common in Sierra Leone	139	45.4
	**Transmission**	**Correct answer**	**%**
5	Hepatitis B can be transmitted through blood transfusion	201	65.7
6	Hepatitis B can be transmitted through unprotected sex	199	65.0
7	Hepatitis B can be transmitted from mother to child	147	48.0
8	Hepatitis B can be transmitted through use of unsafe needles or sharps (e.g., razors and toothbrushes)	203	66.3
9	Hepatitis B can be transmitted through a handshake	247	80.7
10	Hepatitis B can be transmitted through mosquito/insect bites	134	43.8
11	Hepatitis B can be transmitted through coughing or sneezing	106	34.6
12	Hepatitis B can be acquired by eating with or sharing food and utensils with a person with chronic HBV	103	66.3
13	An individual can be infected by both Hepatitis B and HIV	166	54.2
	**Symptoms and sequelae**	**Correct answer**	**%**
14	Hepatitis B infection can lead to yellowing of eyes	167	54.6
15	Hepatitis B infection can lead to swollen belly	123	40.2
16	Hepatitis B infection can lead to liver cancer	146	47.7
17	Hepatitis B infection can lead to cirrhosis (scarred liver)	124	40.5
18	A person can be infected with hepatitis B and not have any symptoms of the disease	134	43.8
	**Prevention and Treatment**	**Correct answer**	**%**
19	There is a safe and effective vaccine to prevent Hepatitis B	106	34.6
20	Hepatitis B can be prevented by using condoms	196	64.1
21	Hepatitis B can be prevented by screening blood before transfusion	199	65.0
22	Hepatitis B can be prevented by washing hands	82	26.8
23	Hepatitis B can be prevented by cooking food	123	49.2
24	Hepatitis B can be prevented by vaccination of uninfected pregnant women	127	41.5
25	Hepatitis B can be prevented by vaccination of newborns	140	45.8
26	Hepatitis B can be cured with medications	82	26.8
27	Hepatitis B can be managed with medications	198	64.7
28	Hepatitis B can be treated by traditional medicine or herbs	154	50.3
	**Stigmatizing attitudes**	**Yes**	**%**
29	Would you have concerns sharing food or utensils with someone who has Hepatitis B?	133	43.5
30	Would you have concerns having casual contact or working with someone who has Hepatitis B?	135	44.1
	**Health-seeking Behaviors**	**Yes**	**%**
31	Are you willing to receive the Hepatitis B vaccine if free of charge?	221	72.2
32	If you got Hepatitis B, would you be willing to take a medication for treatment?	246	80.4
33	If you got Hepatitis B, would you be willing to come to the clinic every 3 to 6 months and let us draw your blood to monitor the HBV?	241	78.8

**Table 3:** Average and maximum score of HBV knowledge based on category.

Knowledge Competence	Mean ± SD	Median (Min–Max)	Maximum Possible Score	% of Participants with Correct Responses
Epidemiology	2.4 ± 1.4	3 (0–4)	4	60.0
Transmission	4.9 ± 2.8	5 (0–9)	9	54.4
Symptoms and sequelae	2.3 ± 2.0	2 (0–5)	5	46.0
Prevention and treatment	4.6 ± 2.9	5 (0–9)	10	46.0
Total score	14.1 ± 8.0	15 (1–27)	28	52.2

**Table 4:** Factors associated with good HBV knowledge (N = 162).

Characteristics	Good HBV Knowledge		Univariate Analysis		Multivariate Analysis	
	Yes	No	Crude Odds Ratio (95% CI)	*p*-Value	Adjusted Odds Ratio (95% CI)	*p*-Value
**Gender**						
Male	72 (44.4)	83 (57.6)	Ref	**0.021**	Ref	0.650
Female	90 (55.6)	61 (42.4)	1.70 (1.08–2.67)		1.21 (0.54–2.71)	
**Age, years**						
<25	47 (29.0)	47 (32.6)	Ref			
25–34	63 (38.9)	64 (44.4)	1.56 (0.74–3.30)	0.241		
35–44	27 (16.7)	17 (11.8)	1.59 (0.78–3.25)	0.207		
≥45	25 (15.4)	16 (11.1)	0.98 (0.41–2.36)	0.971		
**Relationship status**						
Single	102 (63.0)	100 (69.4)	Ref			
Married	49 (30.2)	39 (27.1)	0.98 (0.14–7.10)	0.984		
Divorced	9 (5.6)	3 (2.1)	0.80 (0.11–5.5.91)	0.823		
Widowed	2 (1.2)	2 (1.4)	0.33 (0.03–3.52)	0.362		
**Educational level**						
Secondary or higher	140 (86.4)	105 (72.9)	2.36 (1.32–4.22)	**0.003**	1.18 (0.50–2.79)	0.707
Primary or lower	22 (13.6)	39 (27.1)	Ref		Ref	
**Employment status**						
Employed	82 (50.6)	59 (41.0)	1.48 (0.94–2.32)	0.091	1.71 (0.47–6.14)	0.414
Unemployed	80 (49.4)	85 (59.0)	Ref			
**Monthly earnings**						
Low (<100 US dollars)	79 (48.8)	94 (65.3)	Ref	**0.004**	Ref	0.277
High (≥100 US dollars)	83 (51.2)	50 (34.7)	1.98 (1.25–3.13)		2.04 (0.56–7.7.41)	
**Hepatitis B status**						
Positive	20 (22.0)	3 (8.1)	3.19 (0.89–11.49)	**0.064**	4.41 (1.16–16.77)	**0.029**
Negative	71 (78.0)	34 (91.9)	Ref		Ref	
**HBV vaccination status**						
Vaccinated	29 (17.9)	7 (4.9)	4.28 (1.81–10.08)	**<0.001**	3.30 (1.09–9.94)	**0.034**
Not vaccinated	133 (82.1)	137 (95.1)	Ref		Ref	
**Has a family member, friend or colleague with Hepatitis B**						
Yes	17 (19.3)	10 (27.8)	1.61 (0.65–3.95)	0.300		
No	71 (80.7)	26 (72.2)	Ref			
**Survey setting**						
Community outreach	90 (55.6)	77 (53.5)	1.09 (0.69–1.71)	0.715		
Pharmacy setting	72 (44.4)	67 (46.5)	Ref			

**Table 5:** Factors associated with stigmatizing attitudes towards HBV (N = 151).

Characteristics	Stigmatizing Attitude		Univariate Analysis		Multivariate Analysis	
	Yes	No	Crude Odds Ratio (95% CI)	*p*-Value	Adjusted Odds Ratio (95% CI)	*p*-Value
**Gender**						
Male	67 (44.4)	88 (56.8)	Ref	**0.030**	Ref	0.053
Female	84 (55.6)	67 (43.2)	1.65 (1.05–2.58)		1.62 (0.99–2.65)	
**Age, years**						
<25	35 (23.2)	59 (38.1)	2.38 (1.13–5.03)	**0.023**	1.81 (0.74–4.38)	0.192
25–34	68 (45.0)	59 (38.1)	1.23 (0.60–2.50)	0.577	1.20 (0.55–2.65)	0.6447
35–44	24 (15.9)	20 (12.9)	1.18 (0.50–2.78)	0.711	1.28 (0.50–3.28)	0.607
≥45	24 (15.9)	17 (11.0)	Ref		Ref	
**Relationship status**						
Single	101 (66.9)	101 (65.2)	Ref			
Married	41 (27.2)	47 (30.3)	1.00 (0.14–7.24)	1.000		
Widowed	7 (4.6)	5 (3.2)	1.15 (0.15–8.51)	0.894		
Divorced	2 (1.3)	2 (1.30)	0.71 (0.07–6.92)	0.772		
**Educational level**						
Secondary or higher	133 (88.1)	112 (72.3)	2.84 (1.55–5.20)	**<0.001**	2.43 (1.24–4.76)	**0.010**
Primary or lower	18 (11.9)	43 (27.7)	Ref		Ref	
**Employment status**						
Employed	81 (53.6)	60 (38.7)	1.83 (1.16–2.89)	**0.009**	1.03 (0.47–2.24)	0.949
Unemployed	70 (46.4)	95 (61.3)	Ref		Ref	
**Monthly earnings**						
Low (<100 US dollars)	71 (47.0)	102 (65.8)	2.17 (1.37–3.44)	**<0.001**	1.35 (0.63–2.90)	0.443
High (≥100 US dollars)	80 (53.0)	53 (34.2)	Ref		Ref	
**HBV status**						
Positive	13 (16.5)	10 (20.4)	0.77 (0.31–1.92)	0.571		
Negative	66 (66)	39 (79.6)	Ref			
**HBV vaccination status**						
Vaccinated	22 (14.6)	14 (9.0)	1.72 (0.84–3.50)	**0.133**	1.05 (0.48–2.31)	0.900
Not vaccinated	129 (85.4)	141 (91.0)	Ref		Ref	
**Has a family member, friend or colleague with HBV**						
Yes	17 (22.4)	10 (20.8)	1.10 (0.45–2.64)	0.840		
No	59 (77.6)	38 (79.2)	Ref			
**Survey setting**						
Community outreach	69 (45.7)	98 (63.2)	Ref	**0.002**	Ref	**0.037**
Pharmacy setting	82 (54.3)	57 (36.8)	2.04 (1.29–3.23)		1.74 (1.03–2.93)	
**Good HBV knowledge**						
Yes	97 (64.2)	65 (41.9)	2.49 (1.57–3.94)	**<0.001**	2.10 (1.27–3.50)	**0.004**
No	54 (35.8)	90 (58.1)	Ref		Ref	

## Data Availability

The data presented in this study are available on request from the corresponding author.

## Supplementary Materials

The following supporting information can be downloaded at: https://www.mdpi.com/article/10.3390/healthcare11020177/s1, “Questionnaire on Knowledge, Attitudes and Practices Towards Hepatitis B in Sierra Leone”.
