# Supplementary material for: Assessment of Knowledge, Stigmatizing Attitudes and Health-Seeking Behaviors Regarding Hepatitis B Virus Infection in a Pharmacy and Community Setting in Sierra Leone: A Cross-Sectional Study
Source: Healthcare (Basel). 2023 Jan 6;11(2):177. doi: 10.3390/healthcare11020177 (PMC9859485; doi:10.3390/healthcare11020177)
Supplement: Supplementary file 1 [file healthcare-11-00177-s001.zip › healthcare-2053462-supplementary.pdf]

A Sierra Leone-CWRU Collaboration

Project Title: *Impact of HIV on Hepatitis B Clinical Outcomes in Sierra Leone 2021-2023*

Principal Investigator: George Yendewa, MD, MPH&TM

QUESTIONAIRE ON KNOWLEDGE AND ATTITUDES TOWARDS HBV

Hello. You have been invited to participate in a survey on the knowledge and attitudes with respect to hepatitis B, its prevention, and its treatment. This study is being done by KnowHep Foundation in Freetown, Sierra Leone and Case Western Reserve University in Cleveland, Ohio, USA. Your answer will be important for us to understand what people in Sierra Leone think about HBV.

This is an anonymous study, and your participation is completely voluntary. We will not disclose your answers to anyone else outside our research team. We will do our best to keep your information safe by not writing down your name and using a special code on the questionnaire. We will also keep all questionnaire locked in a filing cabinet at infectious department. If we share your information with other researchers, they will use the same protections.
It will take about 10 minutes to answer the questions. It is OK to choose "don't know" if you don't know the answer. If you have any questions during the survey, you may ask the study coordinator to explain/clarify. You do not have to answer all the questions and you may stop at any time.

If you have any questions after the survey, you may contact Dr. George Yendewa (gay7@case.edu) or Dr. Manal Ghazzawi (knowhepfoundation.sl@gmail.com)

Thank you!

DEMOGRAPHICS AND BASIC INFORMATION

Age: ---

Do you have Hepatitis B infection? Yes No Don't know

Relationship status: Single Married Widowed Divorced

No. of sexual partners in last year: 0 1 2 3+

No. of children: 0 1 2 3+

Education: None Primary school Secondary school College or tertiary

Monthly earnings: < 500,000 Leones >500,000 Leones

HIV Status: Positive Negative Do not know

Family member with Hepatitis B: Yes No Don't know

Family member with HIV: Yes No Don't know

History of blood transfusion: Yes No

History of body piercing or tattoos: Yes No

History of sharp or needle injury: Yes No

History of Hepatitis B vaccination: Yes No

KNOWLEDGE AND RISK ASSESSMENT

A Sierra Leone-CWRU Collaboration
**Project Title:** *Impact of HIV on Hepatitis B Clinical Outcomes in Sierra Leone 2021-2023*
**Principal Investigator:** George Yendewa, MD, MPH&TM

	**Yes**	**No**	**Don't know**
Hepatitis B is caused by a virus			
Hepatitis B can be caused by a curse or evil spirits			
Hepatitis B affects both males and females			
Hepatitis B is common in Sierra Leone			
Hepatitis B can be transmitted through blood transfusion			
Hepatitis B can be transmitted through unprotected sex			
Hepatitis B can be transmitted from mother to child			
Hepatitis B can be transmitted through use of unsafe needles or sharps (e.g., razors and toothbrushes)			
Hepatitis B can be transmitted through a handshake			
Hepatitis B can be transmitted through mosquito/insect bites			
Hepatitis B can be transmitted through coughing or sneezing			
Hepatitis B can be acquired by eating with or sharing food and utensils with a person with chronic HBV			
An individual can be infected by both Hepatitis B and HIV			
Hepatitis B infection can lead to yellowing of eyes			
Hepatitis B infection can lead swollen belly			
Hepatitis B infection can lead to liver cancer			
Hepatitis B infection can lead to cirrhosis (scarred liver)			
A person can be infected with hepatitis B and not have any symptoms of the disease			
Where do you get most of your information about Hepatitis B from?			
• doctor/nurse/healthcare worker			
• TV/radio			
• School			
• Peers/community			

A Sierra Leone-CWRU Collaboration
**Project Title:** *Impact of HIV on Hepatitis B Clinical Outcomes in Sierra Leone 2021-2023*
**Principal Investigator:** George Yendewa, MD, MPH&TM

PREVENTION AND TREATMENT

	Yes	No	Don't know
There is a vaccine for Hepatitis B			
Is the vaccine for Hepatitis B safe and effective?			
Hepatitis B can be prevented by using condoms			
Hepatitis B can be prevented by screening blood before transfusion			
Hepatitis B can be prevented by washing hands			
Hepatitis B can be prevented by cooking food			
Hepatitis B can be prevented by vaccination of pregnant women			
Hepatitis B can be prevented by vaccination of newborns			
Hepatitis B can be cured with medications			
Hepatitis B can be managed with medications			
Hepatitis B can be treated by traditional medicine or herbs			

ATTITUDES

Are you willing to be screened for Hepatitis B?	Yes	No	Don't know
Are you vaccinated against Hepatitis B?			
Are you willing to receive the Hepatitis B vaccine if free?			
Would you have concerns sharing food or utensils with someone who has Hepatitis B?			
Would you have concerns having casual contact or working with someone who has Hepatitis B?			
If you got Hepatitis B, would you be willing to take medication for treatment?			
If you got Hepatitis B, would you be willing to come to the clinic every 3-6 months and let us draw your blood for tests?			

KEY

Each question answered correctly represents 1 point.

Participants who score at least 50% in each section represents sufficient level of knowledge in that section

- **Good knowledge of HBV and risk factors: > 50%**
  Poor knowledge of HBV and risk factors: < 50%

- **Good understanding of HBV prevention and treatment: > 50%**
  Poor understanding of HBV prevention and treatment: < 50%

- **Good attitude towards HBV management: > 50%**
  Poor attitude towards HBV management: < 50%
